# Navigating the Revolutionary Pathway Through Addressing the Challenges in Implementing Dental Blockchain Technology: A Review

**DOI:** 10.7759/cureus.81992

**Published:** 2025-04-10

**Authors:** Mrunali G Gharat, Karishma Ashok, Anupa R Shetty, Prajakta Rao, Amit Patil, Nimisha Nandanan

**Affiliations:** 1 Department of Periodontics and Oral Implantology, Bharati Vidyapeeth (Deemed to be University) Dental College and Hospital, Navi Mumbai, IND; 2 Department of Conservative Dentistry and Endodontics, Bharati Vidyapeeth (Deemed to be University) Dental College and Hospital, Navi Mumbai, IND; 3 Department of Periodontics and Oral Implantology, Shri Vinoba Bhave Civil Hospital, Silvassa, IND

**Keywords:** big data in dentistry, blockchain technology in dentistry, dental blockchain, dental data security, ehr management, electronic health records(ehr), smart contracts in dentistry.)

## Abstract

The integration of blockchain technology into the dental industry has a significant potential to change different aspects, especially patient data management, safety registration, and improving the transparency of transactions. However, the application of dental blockchain faces many challenges, such as technology complexity, obstacles as prescribed, data security issues, and resistance to the changes of stakeholders in the industry. This review article discovers the revolutionary path of blockchain implementation in dental practice by offering these challenges and an overview of feasible solutions. It examines the current status of dental blockchain technology, its potential advantages in improving safety, access, and efficiency, and obstacles that lead to general implementation. Further inspection of the pilot applications and projects in the real world, emphasizing the need for standardized executives, cooperation between stakeholders, and creative approaches to overcome obstacles, is crucial to bridge the existing gap. Finally, the article has a roadmap for future research and development to guide dental experts, decisions, and technologies to navigate the evolutionary landscape of dental blockchain technology successfully.

## Introduction and background

There have been notable advancements and improvements in oral healthcare interventions, technologies, and outcomes. Nevertheless, despite oral health professionals' dedication to patient-centered care, there are significant restrictions and difficulties pertaining to the coverage, accessibility, availability, appropriateness, and affordability of oral health treatment worldwide. Systemic flaws in the model and delivery of oral health services are frequently the cause of the issues. Oral health is essential to overall health and helps those involved in society reach their full potential. According to the Global Oral Health Status Report: Towards Universal, oral diseases are the most common non-communicable diseases, impacting over 50% of the world's population (45%, or 3.5 billion people globally) from early childhood to old age. In American dentistry practices, computers are used. Over the past 30 years, computer use in dentistry offices in the United States has increased. By 1984, 11% of general dentistry practices had computers in their offices. By 2009, that number had risen to over 85% of the 166,000 dental practices in the US. Although there hasn't been much more adoption of electronic dental records (EDRs) by dental offices, there has been a tremendous rise in the use of clinical computing and EDRs by American dentists, particularly in the past 10 years. Schleyer et al. conducted a thorough study through 2006 on the adoption, utilization rates, and attitudes toward clinical computing among general U.S. dentists. They found that 1.8% of dentists were entirely paperless and 25% of dentists used chairside computers [1]. Electronic Health Records (EHRs/HERs), Electronic Health Data (EHD), and Electronic Medical Records (EMR) are digitalized patient records that contain a wide range of medical data, including demographics, laboratory test results, medical histories, and other sensitive patient personal information like credit card numbers and social security numbers [2]. Because there is a risk of health information being leaked to unauthorized parties, outsourcing private health data to the cloud poses a significant privacy challenge for EHRs [3]. Blockchains are decentralized, distributed information repositories protected by a variety of cryptographic primitives. Participants (including payers, providers, and patients) should ideally contribute data to the chain in a safe, verified manner [4]. Blockchain technology has the potential to revolutionize the dental industry by providing secure and efficient data management and patient care solutions [5]. With its ability to provide three-dimensional digital imagery, this technology holds major potential in dentistry, offering a foundation for enhancing dental procedures and addressing future healthcare demands. The application of the metaverse in dental services, encompassing diagnostics, treatments, and education, merits specific attention. Sectors that adopted blockchain in its early stages can serve as excellent models for those that lag behind, as there will be clearer proof of both good impacts and constraints [6]. Given the existing data on blockchain in dentistry, the purpose of this review is to highlight the various obstacles that this unique technology faces in terms of application and resolution.

## Review

Methods

An electronic search was conducted on PubMed, Cochrane, Google Scholar, and Mendeley to identify the article using keywords like EHR management, dental blockchain, and big data in dentistry. The results were transferred to the Mendeley Reference Manager to identify and remove the duplicates and group them after applying the inclusion criteria.

Applications of blockchain technology in dentistry

Because of the distributed ledger's unique features and expanded functionality, the underlying architecture of blockchain has the ability to revolutionize the provision of healthcare, medical, clinical, and life sciences [7]. Blockchain technology eliminates the need for a centralized authority to certify data integrity and ownership, mediate transactions and the exchange of data assets, and certify ownership of information while also enabling secure and fictitious anonymous transactions and agreements made directly between parties. It has important characteristics like immutability, decentralization, and transparency that could potentially address critical concerns in healthcare, including incomplete records at the time of care and challenging access to patients' own health information [8]. A distributed system with ledger capabilities is used to store records in a safe and compatible manner. By eliminating the requirement for a reliable third party to verify data authenticity, document asset ownership, or mediate financial or data transactions, blockchain technology has the potential to completely transform the ways digital aids are exchanged [9].

The applications of blockchain technology, according to studies, are summarized in Table 1.

**Table 1:** Applications of blockchain

Author	Applications
Kashwani et al. [6]	Disaster victims’ identification
Mulchandani et al. [10]	Medical record administration
Hassani et al. [11]	EHRs, dental research, socioeconomics, finances, legal matters
Ostapenko et al. [12]	Feedback platform
Mokhamed et al. [13]	Improve patient confidentiality and privacy, streamline supply chain management, automate services, enhance data management and sharing, and lower fraud and errors
Allareddy et al. [14]	Sharing of data across multiple organizations and stakeholders

Working of blockchain

**Figure 1:**
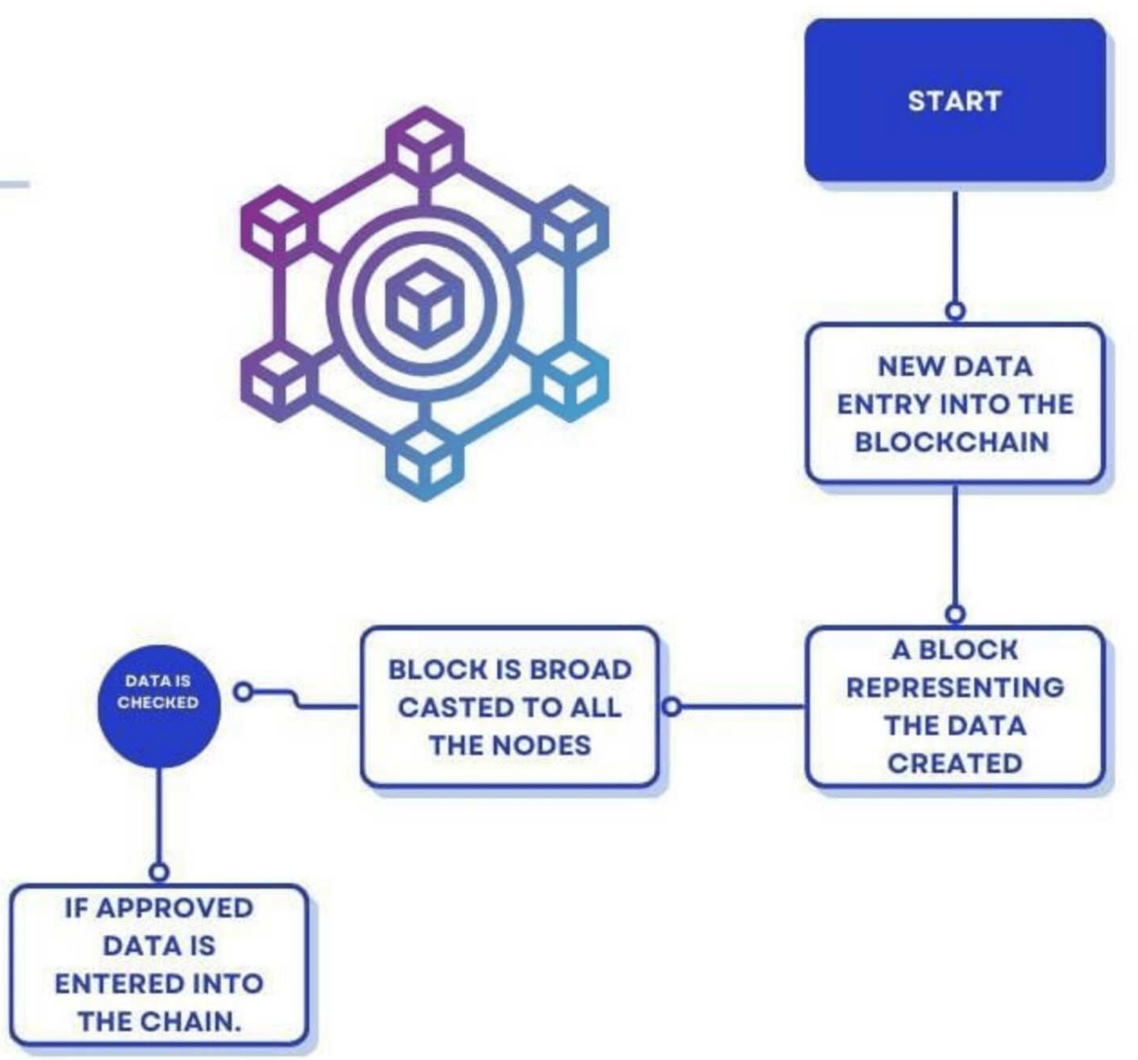
Working of a blockchain The figure is created by authors: Miss Mrunali G. Gharat and Dr. Karishma Ashok

The workings of blockchain are shown in Figure 1.

Types of blockchain

Transactions or information exchanges are carried out via a secure network using blockchain technology. People employ distributed ledger and blockchain technologies in tandem with digital cryptocurrencies. Blockchain is being utilized for private networking and other applications where access and authorization are restricted to members of the network.

Blockchain could be categorized as public blockchain, private blockchain, and hybrid blockchain [15].

*Public* *Blockchain*

With over two hundred use cases revealed globally by governments and other entities, the public sector has emerged as a key application area [16]. A transaction is just considered finalized on the blockchain if both validators concur that it should be recorded there [17]. Notably, modern blockchains present distinct opportunities for business application development and adoption. More specifically, the readily accessible infrastructure of public blockchains allows for the cost-efficient development of applications. Additionally, by utilizing smart contracts, businesses may tokenize off-blockchain assets such as inventory or cash flows. This tokenization has important implications for supply chain management and financing [18].

*Private* *Blockchain*

Because private blockchains are faster, less expensive, and more privacy-focused than public blockchains, they have recently gained greater attention in the industry. The scalability issue, effective consensus mechanism, robust smart contracts, secure access control, replica management, interoperability and compatibility with public blockchains, deployment and feasibility in emerging networks like VANET, edge/fog computing networks, and software-defined networking, and more are just a few of the difficult technological issues that private blockchains have for various industrial Internet of Things (IoT) applications that we should address [18].

*Hybrid* *Blockchain*

IoT systems and the blockchain can be integrated using hybrid blockchain platforms; certain initiatives that do so have various architectural styles [19].

Challenges in implementing blockchain

**Figure 2:**
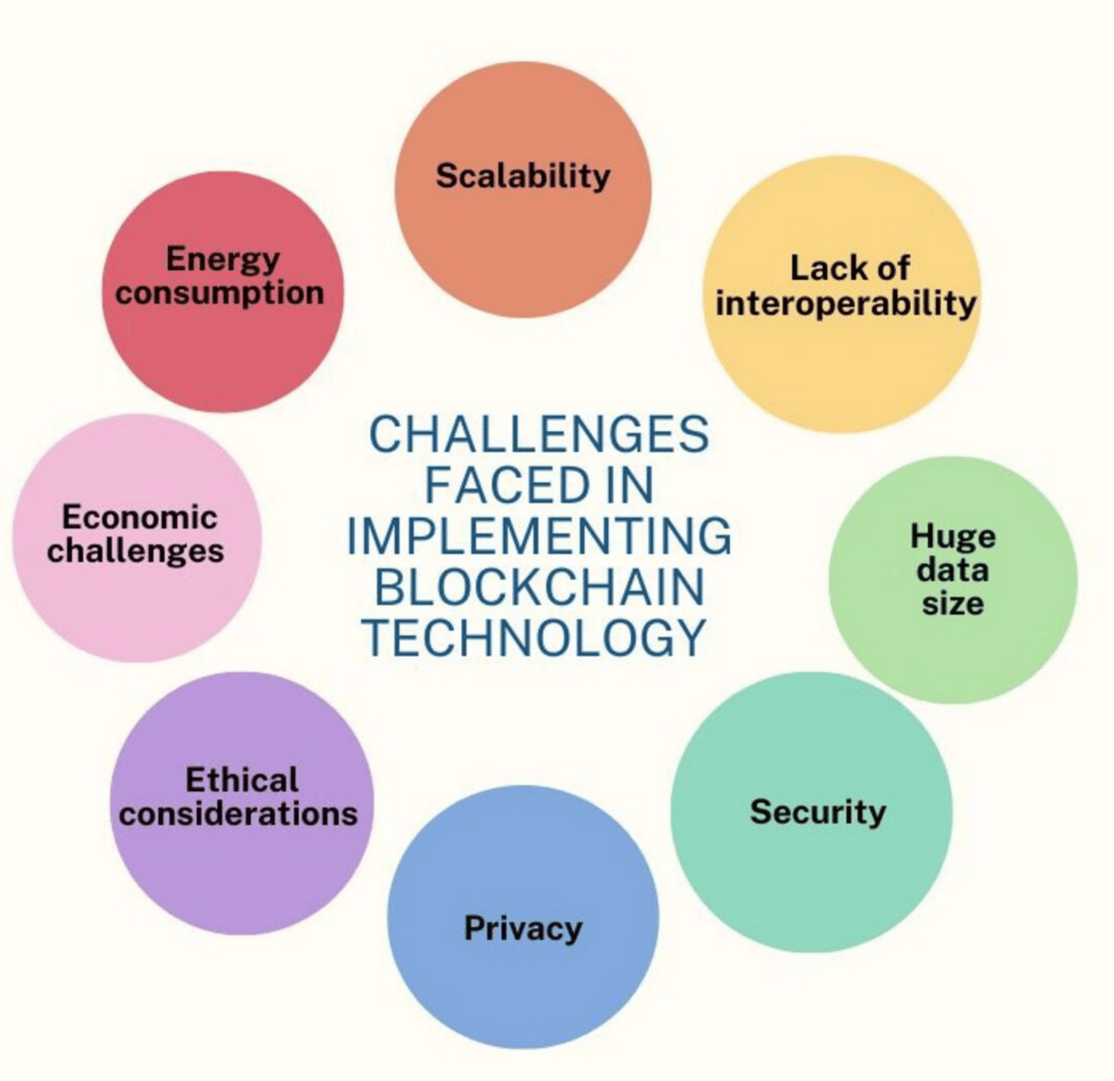
Challenges faced by blockchain technology The figure is created by authors: Miss Mrunali G. Gharat and Dr. Karishma Ashok

The challenges in implementing blockchain technology are shown in Figure 2.

*Energy* *Consumption*

One of the significant drawbacks of blockchain technology is the energy consumption surrounding its application [20].

Scalability

Blockchain technology’s decentralized and immutable data storage has changed several sectors. However typical blockchain networks' scalability issues prevent them from being widely used for large-scale applications [21]. When handling massive transactions, blockchain networks may experience scalability problems. The network may encounter slower transaction times and higher expenses as the quantity of users and data grows [5]. The data can be shared via an off-chain dedicated channel to boost scalability and leverage blockchain technology. The link or even evidence of the data sharing can be stored in the blockchain for auditing and tracking purposes. Intercompany channels are necessary for off-chain data exchange solutions, which adds to the workload for the business in terms of creating and maintaining these channels. Furthermore, the integrity of the data that a business shares cannot be guaranteed by these solutions. For instance, before sharing the data with Company B, Company A may alter the original data to suit Company B's particular needs. The data can be stored and shared on a cloud platform to lessen the load on the participating businesses [22].

*Lack* *of* *Interoperability* 

This leads to fragmented data and inefficient workflows, providing secure and private access to EHRs stored across the system, and saving sensitive data on the cloud, which exposes the system to several risk issues [23]. The majority of suggested solutions have been created independently, lacking a common cryptographic framework or protocol. The inability of solutions operating on various blockchain platforms to connect with one another has resulted in the interoperability issue, which restricts the range of applications. It is anticipated that if the interoperability issue is not resolved promptly, the widespread adoption of blockchains in several domains, including the IoT, may hinder technological progress [24]. Standards will ultimately be essential for ensuring blockchain interoperability and for establishing guidelines for the secure transport and storage of health data [25].

*Size* *of* *Information*

The framework of clinical data is high-volume, high-frequency transactions, and its volume is growing at an exponential rate. The most significant problem with blockchain-stored healthcare data is the enormous amount and size of clinical data. Organizations should agree on a framework for specifying the types, sizes, and formats of data that can be contributed to standardize data stored on the blockchain and control performance. However, this alignment is a hurdle in and of itself because blockchains are collaborative, and standards enforcement and interoperability are real challenges. The most massive issue is instances of blockchain double transactions and ledger fraud. As a result, blockchain technology raises questions regarding wallet and transaction security. When dealing with healthcare data, privacy and security are non-negotiable, and unintended privacy breaches are a real risk [7].

Security

When health records are always in danger of being altered, this is referred to as a security risk [26].

Privacy

According to Materwala et al., privacy pertains to the issue of unobservability, which is also referred to as data leakage, wherein patient health details are utilized without any trace. Significant medical challenges include data sharing, reproducibility, privacy concerns over personal information, and the challenges of enrolling patients in clinical trials [7].

*Ethical* *Considerations* 

Dental practitioners, technologists, policymakers, and patients must collaborate to ensure that these technologies are used responsibly and ethically. Regulatory frameworks must evolve to safeguard patients while fostering innovation, and healthcare professionals must adapt to changing landscapes and embrace new skills and practices [6]. Blockchain-based systems can infringe on patient privacy or potentially compromise the whole network of stakeholders. The public's and individual users' lack of acquaintance with the technology's operation, technical features, or data management advantages is another significant obstacle [27].

*Economic* *Challenges*

Even though decentralization is considered one of the most important characteristics of blockchain, it is not without a cost [28].

Solutions to challenges faced in implementing blockchain in dentistry​​​​​​​

*Security* *and Privacy Issues*

Research efforts have suggested applications using the combined blockchain-cloud (BcC) paradigm to address the scaling challenges caused by the blockchain algorithms and the security and privacy issues prevalent in cloud healthcare systems and to create more reliable solutions for effective patient care. Peer-to-peer networks like blockchain address the problems with the cloud-based strategy. This is due to the blockchain's decentralization, replication, and immutability features. A review of the blockchain-based healthcare system is presented in the literature [26].

Scalability

Sharding has emerged as a workable solution for scalability problems by splitting the blockchain network into smaller, easier-to-manage segments called shards [21]. The main chain's burden can be lessened using off-chain and child-chain techniques. The scalability issue cannot be resolved by simply increasing the block size; the Merkelized Abstract Syntax Tree (MAST) algorithm partially resolves this issue. By separating the signatures using the Segwit approach, less memory will be needed to hold the signatures. A hashing algorithm (Schnorr signature) can be used to create a short signature [29].

Interoperability

One straightforward way for blockchains to communicate with one another is through relay mechanisms, without the assistance of reliable third parties. Under these systems, the blockchains themselves are responsible for giving other blockchains information about themselves, which the receiving blockchain then verifies. Relay-based methods that use an extra component are called relay chains. Validator nodes on the central chain, which facilitates interoperability, are in charge of confirming and [30] carrying out cross-chain transactions. This core chain, which connects the interoperating blockchains, processes all requests for interoperation. During the transaction validation stage of a relay chain solution, the central chain executes its own consensus algorithm [24].

*Huge* *Information Size*

Mahapatro et al. illustrated theoretically that by restricting the number of blocks that an adversary can store along with distributing each segment across several blocks in the cloud, it is better suited for managing massive amounts of data because the storage demand is significantly lower than in traditional architectures. The primary purpose of this strategy is to build a blockchain that can successfully manage big data by reducing storage space by 33%.

The solutions to the huge information size challenge faced by blockchain technology are shown in Table 2.

**Table 2:** Solution to the huge information size challenge faced by blockchain technology

Author	Solution
Mahapatro et al. [30]	Blockchain segmentation
Laouid et al. [31]	A binary matrix-based data representation technique
Arigela et al. [32]	Cloud computing
Kalita et al. [33]	Limit blockchain size by maintaining a minimally shared blockchain allowing new miners to communicate faster
Marsalek et al. [34]	Compressible blockchain architecture using snapshots
Nishida et al. [35]	Information sharing on swarm robotic systems by using blockchain technology
Nadiya et al. [36]	Block summarization and compression

*Ethical* *Considerations*

Solutions to ethical considerations are shown in Table 3.

**Table 3:** Solutions to ethical considerations

Author	Objective
Singh et al. [37]	Balancing transparency with privacy
Scepanovic et al. [38]	Provide a thoughtful study that can assist politicians, healthcare practitioners, and technologists in realizing blockchain’s potential while adhering to ethical norms in healthcare
Agerskov et al. [39]	Outlines a top-down strategy that bases ethical standards for blockchain systems on moral dilemmas
Tang et al. [40]	To get act as a preliminary road map for the investigation of blockchain ethics, as well as to prompt additional discussion and timely awareness-raising on blockchain ethics within the IS community
Dierksmeier et al. [41]	Comprehensive ethical framework. Proper governance and regulation. Focus on prosocial applications, engagement with stakeholders, continuous ethical assessment
Benedetto et al. [42]	While acknowledging that there are several obstacles in the way of possibly putting such a code into practice, the article suggests that ethical codes of conduct be considered for the blockchain business
Kirchschlaeger et al. [43]	Establishing “blockchain code of conduct”
Sharif et al. [44]	Verification of information. Transparency in processes. Immutable records. Encouragement of ethical practices
Kirchschläger et al. [45]	Enhancing transparency and accountability. Addressing data protection and privacy. Mitigating environmental impact. Promoting inclusivity. Regulating unethical uses
Wijesekara et al. [46]	Ethical knowledge sharing can be facilitated by Blockchain by leveraging its security properties, consensus approaches and smart contracts

*Cost* *Effectiveness*

Eliminating extraneous participants during the medical procedure results in significant cost savings [11]. The steps involved in creating a dental blockchain are as follows: an electronic ledger that is dispersed among a network of agents known as validators is called a blockchain. Validators add transactions to the current ledger by batching them into distinct packets called blocks. Thus, the term "blockchain" refers to a virtual chain of transaction records. Blockchain users and validators are different in that although users submit transactions for the blockchain to process, validators decide whether or not those transactions settle. 

Discussion

**Figure 3:**
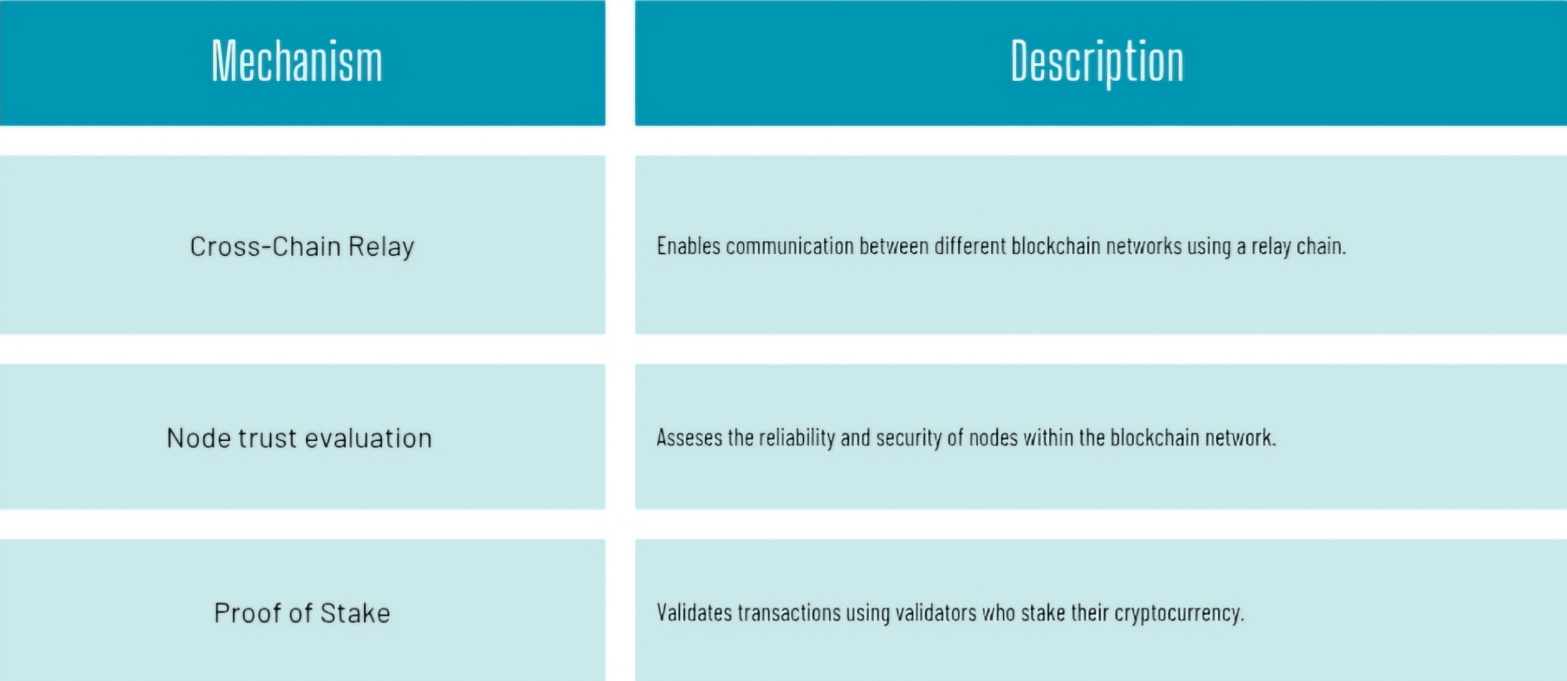
Relay mechanisms The figure is created by authors: Miss Mrunali G. Gharat and Dr. Karishma Ashok

The incorporation of dental blockchain technology into oral healthcare is a promising option for tackling longstanding issues in the industry, notably those related to accessibility, affordability, and data management. As the article points out, while advances in dental care have been made, the ongoing challenges around the management of EHRs continue to be a substantial obstacle to providing optimal patient care [14]. The decentralized nature of blockchain technology provides a secure and efficient means for maintaining EHRs, perhaps leading to better patient outcomes. Guaranteeing that patient data is securely maintained and easily available, helps improve communication among healthcare providers, resulting in more coordinated and effective care [11]. Furthermore, the use of smart contracts within the blockchain can streamline processes, reduce administrative burdens, and ultimately lead to cost savings by eliminating unnecessary intermediaries in medical procedures. Challenges for implementation: despite its potential, the research identifies numerous problems that must be overcome before dental blockchain can be successfully implemented [26]. Energy consumption is a major concern, as existing blockchain models can be resource-expensive. Furthermore, scalability remains a significant concern; as the number of transactions grows, the system must be able to handle them without sacrificing performance. Another challenge is interoperability, which requires different healthcare systems to communicate successfully to enable seamless data flow [24]. To address these difficulties, the article proposes novel alternatives such as merging blockchain and cloud computing to improve scalability and using sharding approaches to manage massive data size. Establishing relay mechanisms for interoperability can also help with smoother integration across platforms. These solutions illustrate the importance of a collaborative approach among healthcare stakeholders in developing a strong framework for dental blockchain. The ethical considerations of using dental blockchain technology cannot be ignored. The paper emphasizes the importance of effective governance in ensuring that technology is used responsibly, and patient privacy is protected. The possibility of data misuse or confidentiality breaches raises serious concerns about responsibility and trust in the system. As a result, it is critical to establish clear norms and legal frameworks to control the usage of blockchain in dentistry. Figure 3 shows relay mechanism.

Limitations of Blockchain

Blockchain technology has certain limitations that hinder its wide usage. The key limitations of blockchain technology include interoperability, scalability, privacy, and energy usage [47]. As the number of health records and network participants increases, blockchain faces scalability challenges. Interoperability arises as different healthcare organizations use blockchain services from different cloud providers, creating interoperability challenges in sharing patient data across systems [26]. Blockchain is excellent at protecting data, but finding a balance between security and real-time data processing efficiency in IoT remains a limitation [48].

Future Directions and Recommendations

Addressing security and privacy flaws, increasing scalability, and strengthening integration with other technologies should be the main goals of future blockchain development. For blockchain to be successfully used in a variety of industries, including healthcare, IoT, and AI, these obstacles must be overcome.

## Conclusions

The probe of dental blockchain technology in oral healthcare suggests disruptive possibilities that could address crucial issues, including accessibility, affordability, and data management. The management of EHRs can be considerably improved by exploiting blockchain’s decentralized and secure nature, resulting in better patient care and more efficient processes. Coordination among the dental, healthcare, and technology industries, the development of standardized frameworks, and consideration of the specific needs of the dental ecosystem are all required to overcome these challenges. Future research must focus on building scalable models, ensuring compliance with healthcare legislation, and enhancing blockchain solutions tailored to dentistry clinics. Dental blockchain technology can achieve its full potential and transform the industry into one that is patient-centric, secure, and efficient by addressing these issues cautiously. To successfully overcome the challenges of implementing dental blockchain technology, this review underscores the importance of ongoing innovation, interdisciplinarity, and patient-centric approaches.
